# Case report: The application of neoadjuvant chemoradiotherapy in anal adenocarcinoma combined with perianal Paget disease involving vulvar skin

**DOI:** 10.3389/fonc.2023.1327173

**Published:** 2023-12-15

**Authors:** Gan-bin Li, Xiao-yuan Qiu, Xiao Zhang, Ning Zhang, Guo-le Lin

**Affiliations:** Department of General Surgery, Peking Union Medical College Hospital, Peking Union Medical College, Beijing, China

**Keywords:** anus neoplasms, Paget disease, extramammary, neoadjuvant therapy, efficacy, case report

## Abstract

Anal adenocarcinoma combined with perianal Paget’s disease (PPD) involving the vulva is rare, and there is no established standard treatment. We present the case of a 69-year-old woman with symptoms of intermittent hematochezia and perianal discomfort for 7 months. Upon examination, we discovered a plaque-like hard mass on the right posterior wall of the anal canal, which extended to encompass the anus and dentate line. The lesion skin also extended forward from the gluteal groove, involving the bilateral labial area. Colonoscopy revealed an extensive protruding lesion on the dentate line, which was confirmed as anal adenocarcinoma (mrT4N0M0). The presence of Paget’s cells in perianal and vulvar skins led to the diagnosis of PPD. The strategy of neoadjuvant chemoradiotherapy (nCRT) followed by radical surgery was then made after multi-disciplinary discuss. The scope and extent of perianal and vulvar disease were significantly diminished after nCRT. The patient underwent laparoscopic abdominoperineal resection and vulvar lesion resection, confirming the diagnosis of anal adenocarcinoma (ypT2N0). No evidence of tumor cells was found in perianal and vulvar skin, indicating a complete response. The patient is regularly monitored without recurrence or metastasis.

## Introduction

Paget disease is characterized by adenocarcinoma localized within the epidermis of the nipple or areola of the breast (1). Extramammary Paget disease (EMPD) is a relatively rare malignancy developed on apocrine-rich skin, such as the vulva, scrotum, and penis, with a reported incidence of 0.1-2.4 patients per 1,000,000 person-years (2, 3). EMPD predominantly presents as a slowly enlarging asymmetrical erythematous plaque, and pruritus is a common symptom (4). The mechanism underlying EMPD develop remain unclear; however, a popular theory posits that primary EMPD arises as an intraepidermal neoplasm originating from the cells of the apocrine gland ducts (5). Owing to its nonspecific clinical presentation and insidious onset, EMPD is often misdiagnosed, resulting in delayed treatment (6).

Perianal Paget disease (PPD) develops near the anus, but in rare cases (~20% of EMPD lesions) can spread across the perineum, genitalia, gluteal canal, and anal canal (7). The perianal region is the second most common site, with 5% of all EMPD lesions originating from the anorectal region (1, 7, 8). The standard-of-care management strategy for PPD is comprehensive, including radiotherapy, local excision, and radical surgery; however, no consensus exists regarding the superiority of local excision and radical surgery, especially in cases of accompanied anal adenocarcinoma (9). Here, we report a rare case of an anal tumor combined with PPD involving the vulvar skin with extensive and massive lesions. Based on our experience with the management of rectal cancer, we decided to apply neoadjuvant chemoradiotherapy (nCRT) followed by radical surgical resection, achieving a satisfactory outcome.

## Case presentation

### Complaints and colonoscopy

**Figure 1:**
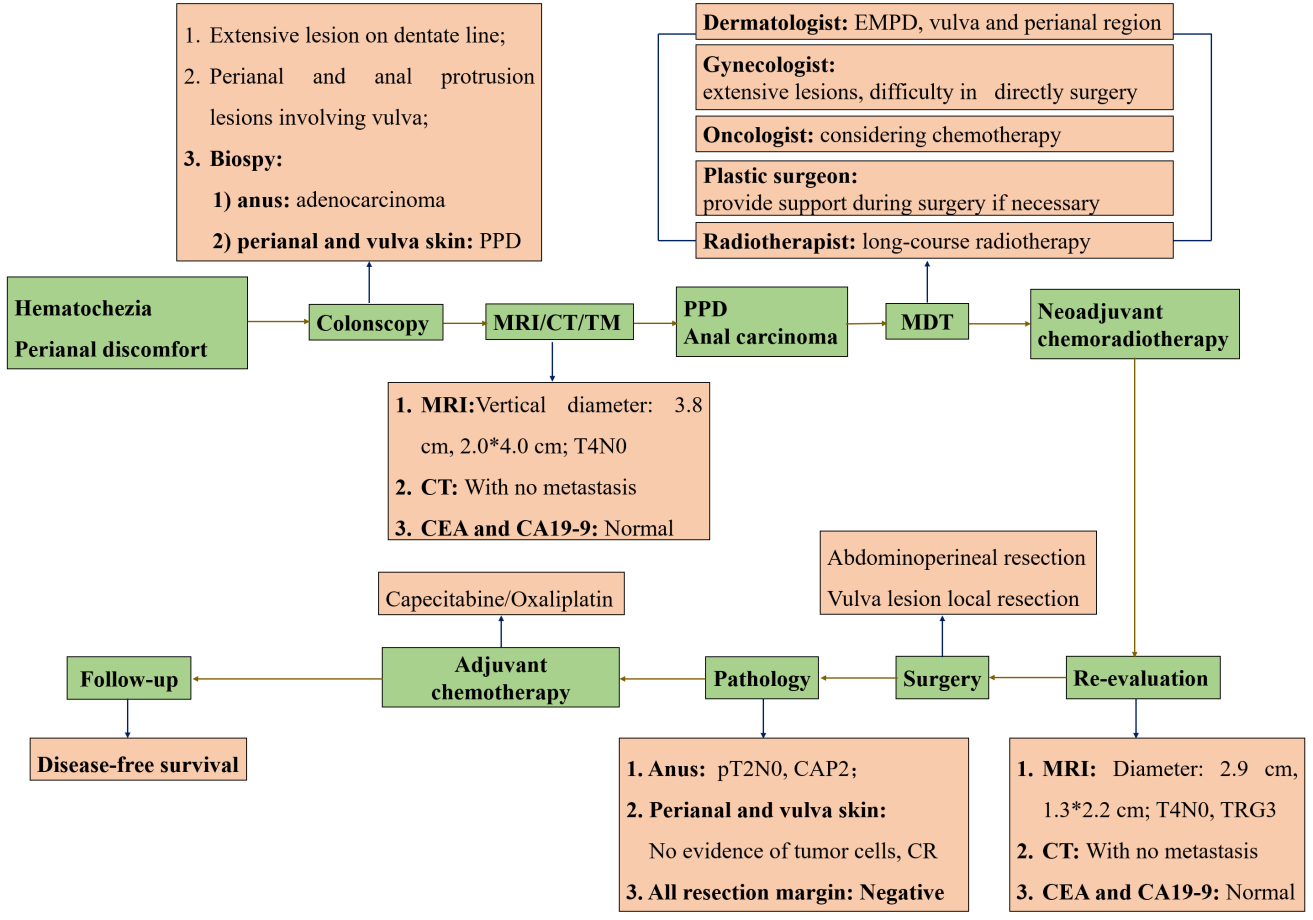
The overview of procedures during the treatment. All procedures marked in green were presented in the order of timelines, all contents marked in orange were supplementary explanation of each procedure. PPD, Perianal Paget disease; EMPD, Extramammary Paget disease; MRI, Magnetic resonance imaging; MDT, Multi-disciplinary treatment; CR, Complete response.

**Figure 2:**
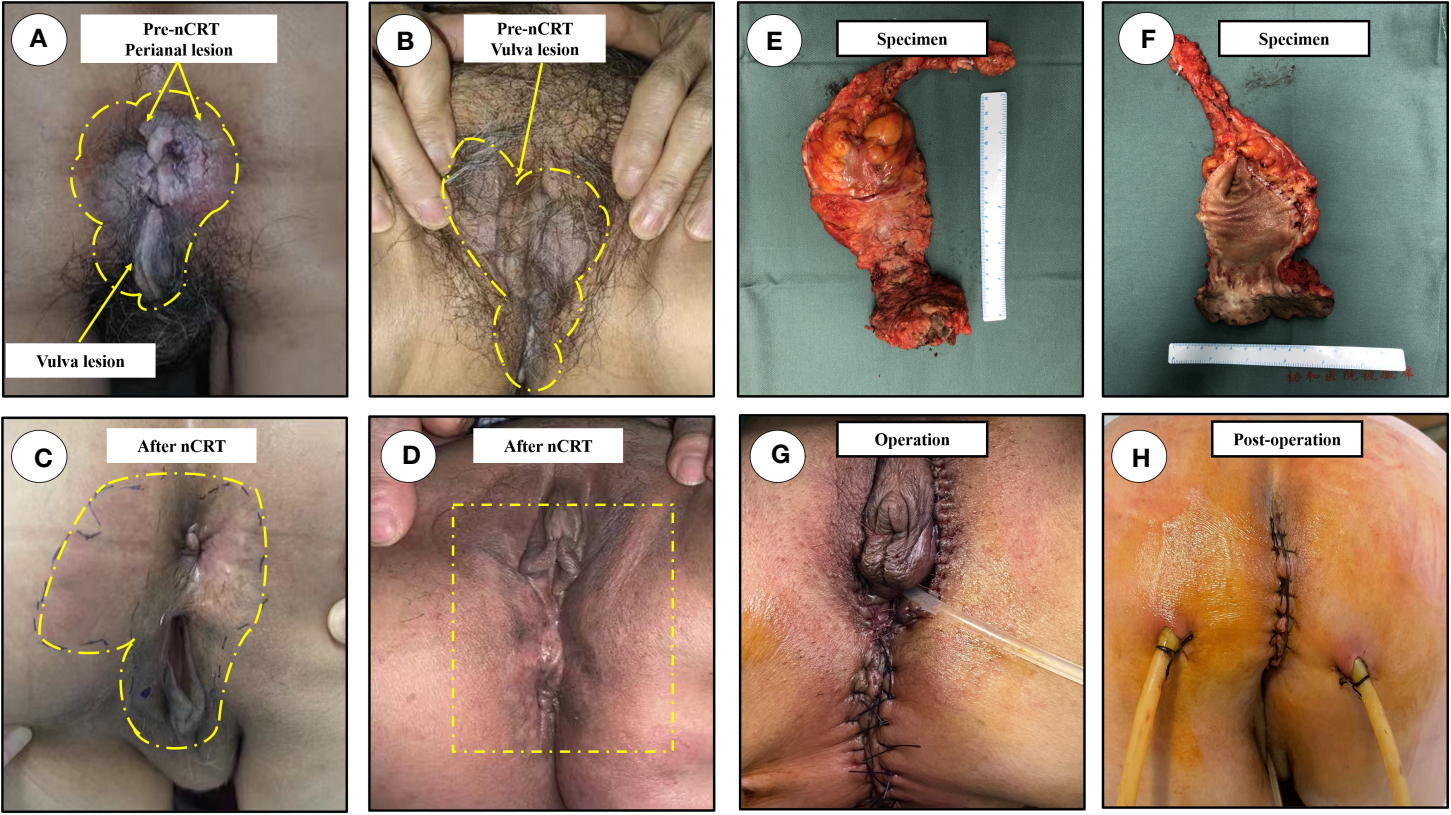
Physical examination. The scope of lesions were marked in yellow dotted line, the most typical lesion was marked in yellow arrow. **(A)** The overall outlook of perianal disease, an obvious mass protruding out of anal canal before nCRT; **(B)** The massive scope of vulva lesions, and the lesion skin before nCRT; **(C)** The overall outlook of perianal region after nCRT; **(D)** the lesions of vulvar region with no macroscopic lesions after nCRT; **(E)** Anterior view of the excised specimen; **(F)** The image of the dissected specimen; **(G)** Images after surgery; **(H)** Perineal incision on postoperative day 9. nCRT, neoadjuvant chemoradiotherapy.

**Figure 3:**
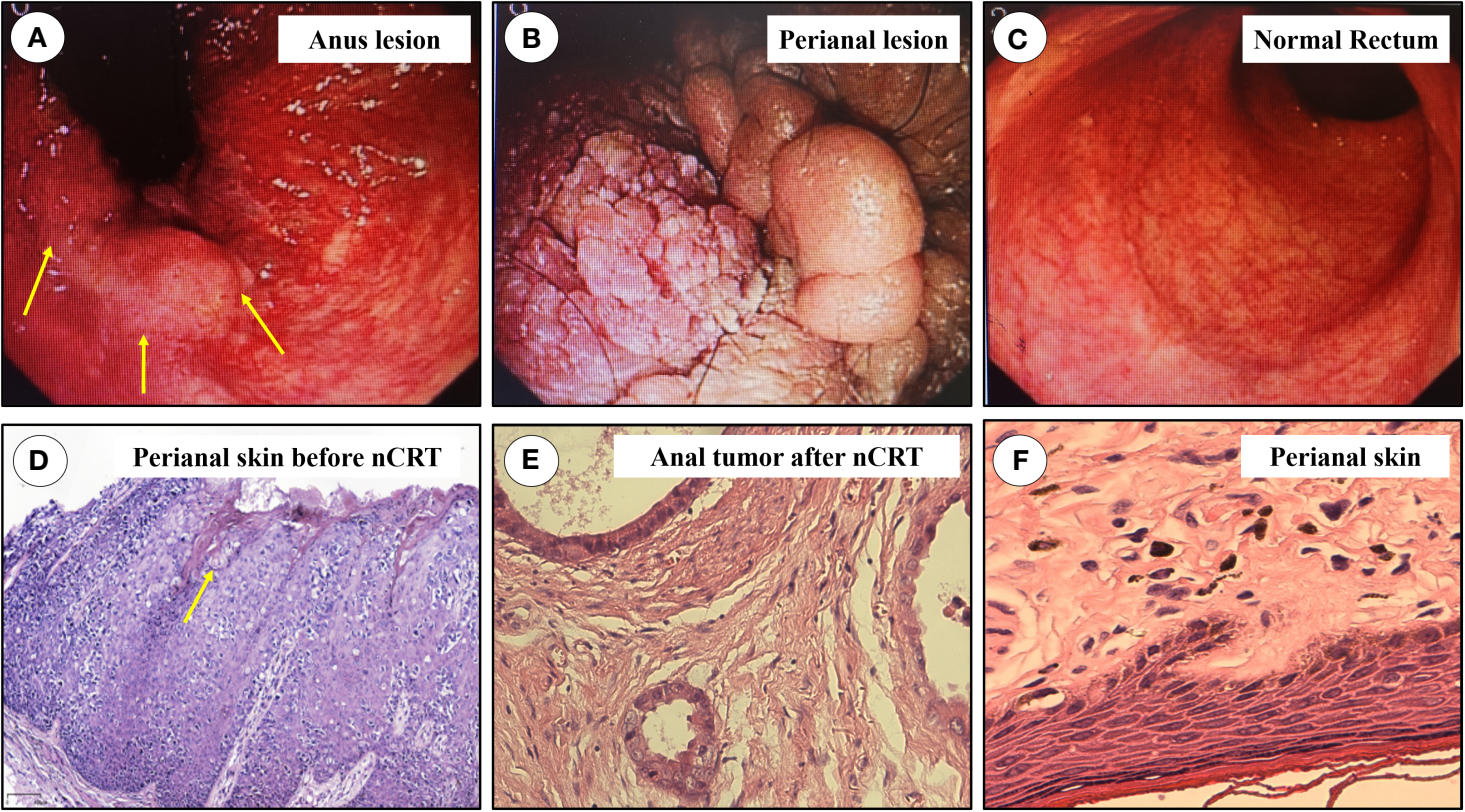
The results of colonoscopy and pathology. **(A)** Anus lesion, protrusion and nodular lesions at anal canal and perianal mucosa, the most typical lesion was marked in yellow arrow; **(B)** The lesions at perianal region; **(C)** The proximal mucosa was normal; **(D)** Pathology of biopsied perianal skin before nCRT, Paget’s like tumor cells could be found, marked in yellow arrow. **(E)** Pathologic result of anal adenocarcinoma; **(F)** Pathologic result of resected perianal and vulva lesions. nCRT, neoadjuvant chemoradiotherapy.

The overall treatment procedure is illustrated in **Figure 1**. A 69-year woman was admitted to our hospital complaining of intermittent hematochezia and perianal discomfort for 7 months. The patient had initially developed intermittent hematochezia, characterized by dark bloody stool, accompanied by perianal pruritus and pain 7 months prior. Upon examination, pigmentation of the local skin and chapped epidermal hyperplasia, as well as a plaque-like hard mass, were found on the right posterior wall of the anal canal, involving the anus and dentate line. The patient’s primary manifestations were changes in defecation habits, including diarrhea and a feeling of urgency during defecation (**Figure 2A**). The aforementioned painful symptoms led the patient to visit the hospital for further examination. A digital colonoscopy (July 14, 2022) revealed an extensive protrudent lesion on the dentate line, and the biopsy results revealed moderately differentiated adenocarcinoma in the anal canal, while papillomatous hyperplasia was characterized by scattered glandular distributed heteromorphic cells with abundant cytoplasm at the lesions around the perianal and vulvar skins, meeting the features of Paget’s cells (**Figures 3A–C**).

### Rectal MRI examination

**Figure 4:**
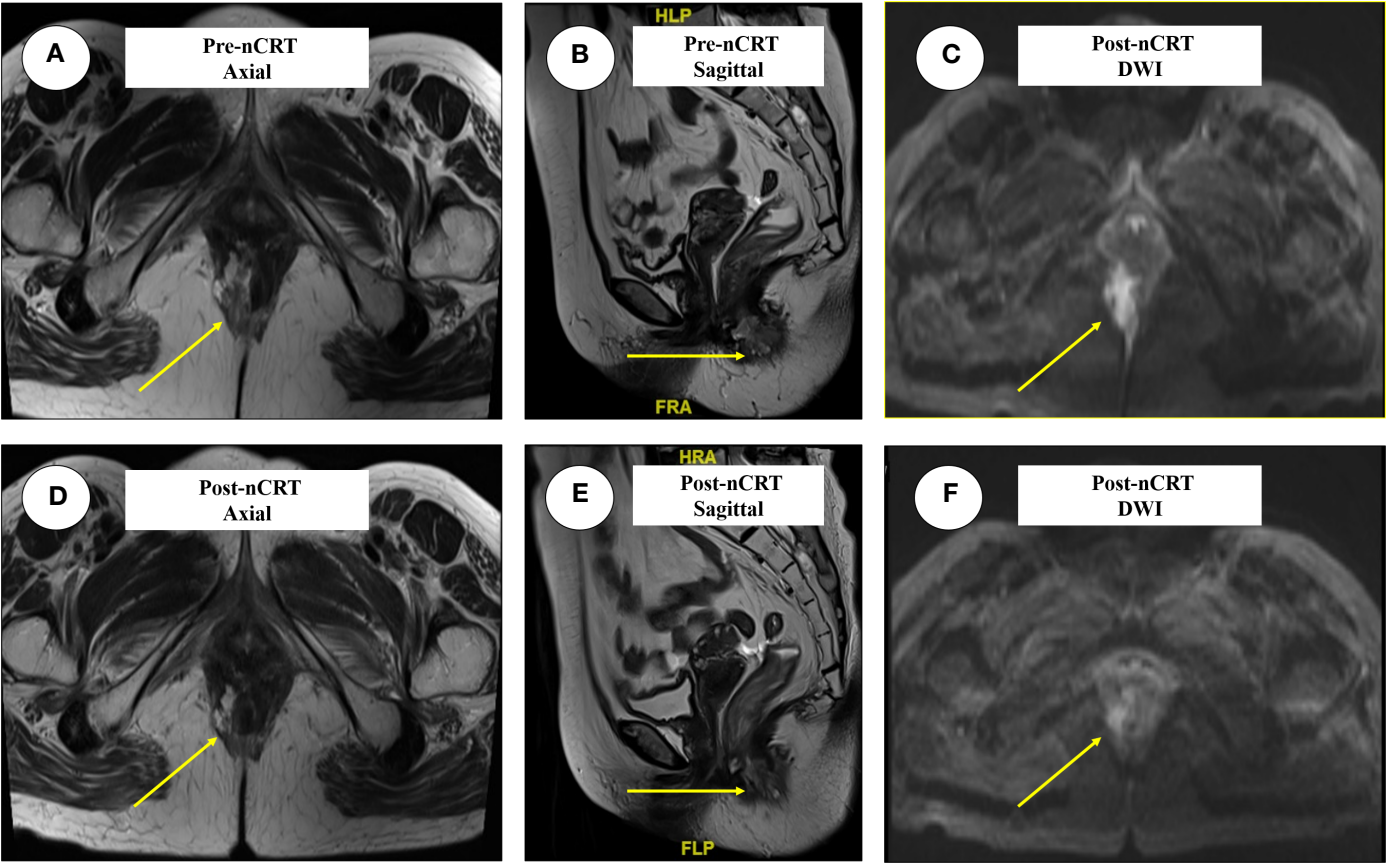
The typical images of anal adenocarcinoma before and after nCRT in three dimensions. The lesions were marked in yellow arrow. **(A)** The axial images before nCRT, the irregular mass protruding into the intestinal cavity; **(B)** The sagittal images before nCRT; **(C)** Lesions presented by DWI before nCRT; **(D)** The axial images after nCRT; **(E, F)** represent the sagittal and DWI lesions after nCRT. nCRT, neoadjuvant chemoradiotherapy; DWI, Diffusion weighted imaging; TRG, Tumor regression grade.

The patient’s typical pathologic features led us to consider the diagnosis of PPD (**Figure 3D**). There were no metastatic lesions in the liver or lungs, and tumor markers were normal. An irregular mass protruding into the intestinal cavity was observed from the 6 to 12 o’ clock positions in the entire section of the anal canal, detected by rectal MRI. The maximum section was 4.0 cm * 2.0 cm. The lesion protruded from the external anal margin, the involved anal canal became stenotic. The anterior edge of the lesion involved the posterior wall of the vagina and was staged as mrT4N0M0 (**Figures 4A–C**).

Physical examination revealed that local skin pigmentation and chapped epidermic hyperplasia could be found on the right side of the anus with a range 2.0×3.0 cm in scope, while a plaque-like hard mass (~1.5 cm in diameter) was observed on the right posterior wall of the anal canal, involving the anus and dentate line. The lesion skin developed from the gluteal groove forward involving the bilateral area of the labia (**Figures 2A, B**).

### Multi-disciplinary discussion

Considering that diagnosis of anal adenocarcinoma combined with PPD involving the vulvar skin, we organized a multidisciplinary panel to determine the optimal strategies. The dermatologist consulted the pathological sections of the perianal skin again, which showed heterotypic epithelial cells visible in the basal layer and epidermis, accompanied by a slightly stained cytoplasm. PPD was considered firstly when combined with immunohistochemical results (positive for CK7/CK20/CEA/PAS). In addition, the scope of the vulvar lesions was determined using fluorescence diagnostic technology and biopsy, which revealed that the epidermal spinous layer was hypertrophic, with a large number of Paget-like cells with obvious atypia. The gynecologist held the view that the chapped and depigmented skin lesions developed from the perianal area to the bilateral regions of the labia, radical surgery was difficult. Although the diagnosis was clear, the oncologist believed that the lesions involved a large area and that direct surgery might be difficult, and neoadjuvant radiotherapy concurrent with capecitabine might therefore be the optimal choice. The plastic surgeon was recruited to assist in the removal and repair of soft tissue lesions, if necessary.

### NCRT and subsequent re-evaluation

A strategy was chosen to administer nCRT followed by radical surgery. The patient received 25 fractions of radiotherapy, followed by 2 cycles of oral capecitabine between October 4, 2022, and November 8, 2022. The treatment course was well-tolerated without any notable discomfort (**Figure 1**).

Re-evaluation by MRI revealed an irregular mass protruding into the cavity of the anal canal. The maximum cross section was about 1.3 cm × 2.2 cm, the solid component of tumor mass was significantly reduced, and mucus signals were visible in the anal canal. The application of nCRT effectively reduced both the volume and diameter of the tumor mass to an extremely diminished status, with MRI indicating a tumor regression grade reaching TRG 3 (**Figures 4D–F**).

Physical examination revealed scattered erythematous changes on the perianal skin, although no eminent lesions observed on the perianal skin. Digital rectal examination (thoraco-knee position) revealed a hard mass on the right posterior wall of the anal canal under the dentate line, which was significantly decreased compared with the pre-neoadjuvant period; lesions on the bilateral labia were also significantly improved, with no obvious pigmentation or chapped skin (**Figures 2C, D**).

### Surgery and follow-up

The patient was subsequently scheduled for laparoscopic abdominoperineal resection combined with vulvar lesion resection on February 3, 2023, the resected specimens of anal tumor along with perianal lesions were displayed in **Figures 2E, F**. The surgery was successful, and the patient was discharged from the hospital on postoperative day 10 (**Figures 2G, H**). Pathological review of the resected specimens revealed that the anal mass was a moderately differentiated adenocarcinoma staged as ypT2N0, and the tumor regression grade was reaching CAP 2 (**Figure 3E**), there was no evidence of tumor cells in the resected perianal and vulvar lesions (**Figure 3F**), Immunohistochemistry indicated positivity for CK20 (+), CDX-2 (+), MUC2 (+), GCDFP-15 (+), and negative CK-7. Follow-up is currently ongoing, and no evidence of recurrence or metastasis has been observed.

## Discussion

EMPD is a malignancy originating from epithelial cells, which is predominantly distributed in the perineum, external genitalia, and other apocrine-rich sites. These lesions are generally confined to the epidermis, dermis, or subcutaneous soft tissue, later transforming into invasive lesions through recurrence and metastasis (8). EMPD is mostly a single-organ disease, and only 4% of cases are complicated by multiple lesions (10).

The incidence of EMPD is low, and its clinical manifestations lack specificity, mainly presenting as erythema, erosion, ulcers and hyperpigmentation (8). Multi-point full-layer puncture is an important diagnostic method for EMPD. In EMPD, single cells or clusters of Paget cells can be arranged in the epidermis, dermis, or subcutaneous tissues. Paget cells are larger than keratinocytes, and their cytoplasm is more pale or granular (11). Immunohistochemistry also contributes to EMPD diagnosis. Positivity for keratin CK7/CK20 and CEA and negativity for SOX10 are suggestive of EMPD, whereas positivity for CK20 and CDX2 suggests the possibility of secondary EMPD (12, 13). In addition, site-specific tumor markers are also helpful for differential diagnosis, such as positivity for GCDFP15, indicating the origin of the genital system, and positivity for CDX2 and CK20, which may refer to perianal lesions, as presented in this case (14). Ultrasonography, colonoscopy, magnetic resonance imaging (MRI), computed tomography (CT), and other examinations can clarify and differentiate between the diagnosis (1, 8, 11).

Radical surgery is a vital treatment for primary EMPD, and the essential principle is to ensure that all surgical margins are negative. Local or extended local resection or Mohs minimally invasive surgery can also be performed; however, the postoperative recurrence rate can reach as high as 30% (13). Conservative treatment or local radiotherapy can also be used as alternative treatments. Adjuvant chemotherapy, targeted therapy, and immunotherapy may also be used to manage metastatic EMPD (15). For secondary EMPD, a multidisciplinary collaboration is required to develop precise treatment plans.

Whether nCRT can be applied in the treatment of invasive PPD, particularly when accompanied by simultaneous anal canal adenocarcinoma, remains controversial (2, 16). In this case, anal adenocarcinoma was combined with PPD that invaded the vulva, and the lesions were diffuse and involved a large area between the perianus and labia. If surgical resection is chosen for the first time, combined abdominoperineal resection and extended resection of the vulva may be mandatory, which is extensive and traumatic; further, flap transplantation and vulvar reconstruction may also be required for radical purpose (17). After a multidisciplinary consultation, we decided to gain experience with the comprehensive treatment of mid-to-low locally advanced rectal cancer using neoadjuvant long-course radiotherapy, followed by concurrent single-agent oral capecitabine for a total of two cycles.

This study has certain limitations. Firstly, in the process of assessing the treatment efficacy after neoadjuvant chemoradiotherapy, there was no subsequent biopsy of the perianal skin and the lesion site in the perineum. Confirming complete remission of perianal and perineal lesions before surgery could have further reduced the extent of the surgical procedure. Additionally, this study only reports one-year postoperative survival outcomes, which represents a relatively short follow-up period. Long-term survival prognosis for patients should be tracked more extensively in the future.

The application of nCRT has two purposes: first, the sizes and scope of lesions can be reduced, in some cases even achieving complete clinical response; second, it can achieve a reduction in tumor size and stage through chemoradiotherapy, as well as increasing tumor local control and eradication of micrometastasis to reduce the possibility of local recurrence and distant metastasis (16, 18). In the present patient, after nCRT, the lesions in the perianal region were significantly reduced, no clear swelling lesions were found around the anus after treatment, and the scope of vulvar lesions was also diminished compared to that before treatment (18). Postoperative pathological results confirmed that after nCRT, no tumor cells could be found around the anus and vulva, and the overall treatment effect was promising. During surgery, radical resection was ensured by repeated inspection of the incisal margin. The resection scope of vulvar lesions was relatively limited, and a one-stage suture was feasible, avoiding flap transplantation and vulvar reconstruction.

## Data availability statement

The original contributions presented in the study are included in the article/supplementary material. Further inquiries can be directed to the corresponding author.

## Ethics statement

The studies involving humans were approved by the Ethics Committee of Peking Union Medical College Hospital (No. JS-1296). The studies were conducted in accordance with the local legislation and institutional requirements. The participants provided their written informed consent to participate in this study. Written informed consent was obtained from the individual(s) for the publication of any potentially identifiable images or data included in this article.

## Author contributions

GL: Conceptualization, Data curation, Formal analysis, Investigation, Methodology, Project administration, Software, Visualization, Writing – original draft. XQ: Conceptualization, Data curation, Formal analysis, Methodology, Project administration, Writing – review & editing. XZ: Conceptualization, Data curation, Formal analysis, Methodology, Resources, Writing – review & editing. NZ: Funding acquisition, Investigation, Methodology, Project administration, Resources, Software, Supervision, Writing – review & editing. GL: Funding acquisition, Investigation, Methodology, Resources, Software, Supervision, Validation, Visualization, Writing – review & editing.
